# Recycling Plastics from WEEE: A Review of the Environmental and Human Health Challenges Associated with Brominated Flame Retardants

**DOI:** 10.3390/ijerph19020766

**Published:** 2022-01-11

**Authors:** Cecilia Chaine, Andrew S. Hursthouse, Bruce McLean, Iain McLellan, Brian McMahon, Jim McNulty, Jan Miller, Evi Viza

**Affiliations:** 1 Restructa Ltd., North Newmoor Industrial Estate, Irvine KA11 4JU, UK; brian.mcmahon@restructa.co.uk (B.M.); jim.mcnulty@restructa.co.uk (J.M.); 2 School of Computing, Engineering & Physical Sciences, University of the West of Scotland, Paisley PA1 2BE, UK; andrew.hursthouse@uws.ac.uk (A.S.H.); iain.mclellan@uws.ac.uk (I.M.); evi.viza@uws.ac.uk (E.V.); 3 CCL (North) Ltd., Meadowhead Industrial Estate, Irvine KA115AU, UK; bruce.mclean@cclnorth.com; 4 School of Health & Life Sciences, University of the West of Scotland, Paisley PA1 2BE, UK; jan.miller@uws.ac.uk

**Keywords:** WEEE, brominated flame retardants (BFR), electrical and electronic equipment, waste, WEEE plastics

## Abstract

Waste electrical and electronic equipment (WEEE) presents the dual characteristic of containing both hazardous substances and valuable recoverable materials. Mainly found in WEEE plastics, brominated flame retardants (BFRs) are a component of particular interest. Several actions have been taken worldwide to regulate their use and disposal, however, in countries where no regulation is in place, the recovery of highly valuable materials has promoted the development of informal treatment facilities, with serious consequences for the environment and the health of the workers and communities involved. Hence, in this review we examine a wide spectrum of aspects related to WEEE plastic management. A search of legislation and the literature was made to determine the current legal framework by region/country. Additionally, we focused on identifying the most relevant methods of existing industrial processes for determining BFRs and their challenges. BFR occurrence and substitution by novel BFRs (NBFRs) was reviewed. An emphasis was given to review the health and environmental impacts associated with BFR/NBFR presence in waste, consumer products, and WEEE recycling facilities. Knowledge and research gaps of this topic were highlighted. Finally, the discussion on current trends and proposals to attend to this relevant issue were outlined.

## 1. Introduction

Whether it is to facilitate daily activities, provide comfort, or luxury, electrical and electronic equipment (EEE) have become an essential part of most activities carried out in modern societies. From the use of refrigerators and washing machines, to televisions, mobile phones, and computers, EEE dominates our daily lives. This equipment has a set of associated features, including short lifespans, rapid changes in technologies, and limited repair options for reuse, which result in steadily increasing volumes of waste EEE being generated, mainly in developed countries. Worldwide, generated volumes went from 41.8 Mt in 2014 to 53.6 Mt in 2019, and with a projected annual growth rate of 2 Mt, volumes could reach 74.7 Mt in 2030 [1].

In Directive 2012/19/EU, the European Union has defined the waste of electrical or electronic equipment (WEEE) as electrical or electronic equipment that is waste within the meaning of Article 3 (1) of Directive 2008/98/EC, including all components, sub-assemblies and consumables which are part of the product at the time of discarding. The directive further defines “electrical and electronic equipment” or “EEE” as equipment which is dependent on electric currents or electromagnetic fields in order to work properly and equipment for the generation, transfer, and measurement of such currents and fields and designed for use with a voltage rating not exceeding 1000 volts for alternating current and 1500 volts for direct current [2].

This equipment contains hazardous components, making WEEE a hazardous waste, and even though highest per capita volumes are generated in Europe and Oceania (16.2 and 16.1 kg per capita, respectively [3]), their management is currently a matter of global concern. This is because, promoted by the valuable extractable resources found in WEEE, a high proportion of these wastes are illegally exported to Africa and Asia where they are treated by informal workers under inappropriate conditions from environmental, health, and safety points of view. Consequently, the proper handling, treatment, and disposal of WEEE is the key to achieving its sustainable management, minimizing their potential adverse health and environmental effects, and maximizing their value.

Materials found in WEEE mainly include ferrous and non-ferrous metals, glass, and plastics, the latter accounting for approximately 30% of all WEEE volume in weight generated per year [4]. Furthermore, different types of EEE contain different types of plastics, with acrylonitrile butadiene styrene (ABS), polypropylene (PP), high impact polystyrene (HIPS), and polycarbonate (PC)/ABS blends the most frequently found. Other types of polymers found in WEEE are PU (polyurethane), PE (polyethylene) and PVC (polyvinyl chloride).

As a result of the challenges associated with the complexity of these plastic mixtures, and the limited existing technologies for their adequate sorting, it is estimated that approximately 40 to 50% of captured plastics in WEEE are not being properly recycled [3]. Adding to these low recyclability rates is the presence of hazardous components found in WEEE plastics, including potentially toxic elements (i.e., lead, cadmium, mercury) and flame retardants. 

The circulation of electric currents, heating of internal components, together with the inherent flammability of most plastics and the widespread use of EEE in households and offices, mandates the addition of flame retardants in order to comply with flammability standards. Flame retardants (FRs) include halogenated compounds (organo-halogen flame retardants) with chlorinated and brominated FRs, amounting to 1% and 55% of global use, respectively, according to reports from 2018 [5]. Furthermore, flame retardants are commonly used in combination with antimony trioxide (Sb_2_O_3_) as a synergist with the Sb consumption for this use, accounting for approximately half of its total production [6,7].

According to the Basel Convention, Sb-containing waste must be classified as hazardous. In Europe and the UK, waste containing Sb should be classified under various regulations. For instance, the European Chemical Classification, Labelling and Packaging Regulations establish that any waste containing Sb_2_O_3_ at concentrations higher than 0.1% *w*/*w*, is to be classified as hazardous. As Sb_2_O_3_ is the only Sb compound used as a synergist of halogenated FRs, a total Sb concentration limit of 8400 mg/kg can be considered, making its determination reasonably straightforward. Therefore, even though its presence represents a challenge in WEEE plastic treatment, Sb exceeds the scope of the present review, which is focused on the challenges associated with FR presence. It is, however, important to highlight that, to date, no comprehensive systematic and quantitative studies for the characterization of the presence of Sb in WEEE have been carried out. 

EEE plastics contain chemicals, such as fillers or additives, so that the items meet technical standards, such as those for flammability for which flame retardants like brominated flame retardants are included. These are synthetic additives with a very high efficiency for retarding flames and are comparatively cheaper than other flame retardants. One of the main reasons behind the low recycling rates of WEEE plastics, is their FR content. The most technically relevant brominated flame retardants (BFRs) are polybrominated diphenyl ethers (PBDEs), tetrabromobisphenol A (TBBPA), and hexabromocyclododecane (HBCDD), which due to their characteristics of being additive BFRs and easily leach from the polymeric matrix, were classified as persistent, bio accumulative and toxic to the environment. This will be discussed in more depth in the following sections.

Brominated flame retardants have been long used as additives or reactive FRs since the 1980s in the manufacture of EEE [8], and although their application has been greatly reduced since 2009 after being listed and are thus restricted by the Stockholm Convention, they continue to be found in WEEE at high concentrations. 

Octa-BDE and deca-BDE are among the most frequently used BFRs in electrical and electronic equipment. Being lipophilic and persistent, these compounds can concentrate in animal fat, having potential harmful effects on human health due to their bioaccumulation, biomagnification, long-range transport potential, and their impact on the activity of certain hormones [9].

Although the production of penta- and octa-BDEs has been stopped in many developed countries, the concentrations of penta- and octa-BDEs detected in humans have increased. For example, in Australia, although the import of these BFRs was stopped in 2005, the concentrations reported in humans are higher than those determined in Europe or Asia. However, they do not exceed the human concentrations determined in the United States [10].

The most common routes of human exposure to BFRs are through ingestion or inhalation of dust in enclosed spaces or workplaces, and dietary ingestion. As with a variety of other pollutants, exposure to BFRs in the occupational environment can be much higher than environmental exposure. In fact, some of the highest BFR exposures that have been determined were in offices and WEEE recycling facilities [11].

Considering the complexity associated with the proper handling of WEEE plastics, due to the presence of specific chemical compounds, it is essential that we evaluate the potential effects that such hazardous substances may have on human health, which will ultimately allow monitoring and the definition of the toxicity limits of plastics and, thus, establish environmentally safe handling and treatment processes. 

Numerous studies have been carried out over the last decade to characterize the toxicity of WEEE plastics, e.g., [12,13,14,15,16,17]. While these studies have provided valuable information on the composition of WEEE plastics, they do not discuss potential human health consequences for either recycling site workers or people in the communities surrounding recycling sites [18].

Through the establishment of concentration limits for hazardous BFRs in certain consumer products, there has been a decrease in the use of these BFRs worldwide, which has been accompanied by an increase in the use of novel BFRs (NBFRs) for their replacement. The most common NBFRs are DBDPE (decabromodiphenyl ethane) and TBBPA-DBPE (tetrabromobisphenol A-bis (2,3-dibromopropyl ether)) which are used to replace deca-BDE, octa-BDE and TBBPA, respectively. A review conducted by Xiong et al. [19] gathered the latest information on the presence and distribution of NBFRs, in particular DBDPE BTBPE, HBB, PBEB (pentabromoethylbenzene), and PBT (polybutylene terephthalate) in biotic and abiotic environments, and identified their human exposure and toxicity. The data collected show that most NBFRs do not pose significant risks to the environment, but more data from different species are required to better understand their health impacts. It was also demonstrated that NBFRs are ubiquitous in different matrices and their concentrations are generally higher in WEEE recycling sites compared to other locations. In terms of toxicity, it was shown that several NBFRs can cause adverse effects through different modes of action, such as endocrine disruption. 

Endocrine disruptors (defined by the World Health Organization as “exogenous substances or mixtures that alters function(s) of the endocrine system and consequently causes adverse health effects in an intact organism, or its progeny, or (sub) populations”) are xenobiotics and it is hypothesized that the increase in the number of cases of people presenting conditions linked to their adverse health effects, such as neurodegenerative diseases, thyroid dysfunction, diabetes, and infertility, is due to an increase in the concentrations of endocrine disruptors to which people are currently exposed. However, many authors have evidenced that proposed links between health effects and endocrine disruptors are not robust enough and, therefore, further research is required. In a recent publication, Pironti et al. [20] presented a review on endocrine disruptors found in different matrices (water, animals, and human), as well as analytical methods for their detection

To the best of the authors’ knowledge, no reviews have been published on challenges associated with recycling plastics from WEEE, which include legal, industrial, environmental, and human health aspects. Therefore, it is necessary to create a collective component of information in the novel emerging research area of WEEE plastic recycling associated with BFRs. Based on Cronin et al. [21], an extensive survey of legislation and literature was performed on the latest regulations, WEEE plastic sorting technologies, BFR occurrence, and recent findings on their impacts on the environment and health. The most relevant methods of existing industrial processes for determining BFRs and their challenges were identified. BFR occurrence and substitution by NBFRs was overviewed. An emphasis was placed on reviewing the health and environmental impacts associated with BFR/NBFRs presence in waste, consumer products, and WEEE recycling facilities. The knowledge and research gaps on this topic were highlighted. Finally, the discussion on current trends and proposals to attend to this relevant issue were outlined. 

## 2. Management of WEEE Plastics Containing BFRs: Current Trends and Challenges

WEEEs contain various resources, including precious metals (i.e., gold, platinum, copper, silver), as well as other valuable metals, such as iron and aluminum. These high-demand materials make WEEE economically attractive to both informal and formal recyclers. However, their management in the informal sector poses a threat to both the health of the people involved and the surrounding ecosystem due to the presence, generation, or use of toxic substances, including BFRs. This is a worrying matter, particularly in middle and low-income countries where WEEE is usually managed under poor conditions, causing serious health impacts on workers and children who are usually linked to the activity as workers themselves or by playing close to where WEEE is processed [22]. 

It is estimated that around 2.6 million tons of WEEE plastics are currently produced annually in Europe. The mass of this collected material is limited to 700 kt/year of which only approximately 30% (200 kt/year) can be sent for recycling due to current processing capacities. Management of the remaining 500 kt/year of recovered plastics is mainly by incineration for energy recovery or fuel replacement in cement kilns (44%), and landfilling (11%) [23]. It is predicted that by 2050 recycling will increase to 50%, energy recovery to 44%, with landfill being the least selected option decreasing to a projected 6% [1]. 

Two of the main reasons behind the current low recycling rates are the mix of different polymers and the presence of additives, such as flame retardants. It is estimated that 9% of the total volume of WEEE plastics generated per year (234 kt/year) contain BFRs of which only a small fraction corresponds to restricted BFRs, such as octa-BDE or deca-BDE. Furthermore, because of restrictions imposed on their use, concentrations are rapidly, and steadily decreasing (octa-BDE use was restricted in 2003 and deca-BDE in 2008). However, given that EEE is estimated to have a useful life of approximately 12 years, EEE containing these compounds is currently being disposed of [3].

### 2.1. Brominated Flame Retardants (BFRs): Context

BFRs can be reactive or additive components. Table 1 presents characteristic and examples for each type (based on [24]). Additive FRs are considered to be more unstable and volatile than reactive FRs, as a result of their covalent bonds with the polymer matrix.

Polybrominated diphenyl ethers (PBDEs) are a noteworthy group of additive BFRs commonly found in plastics, foams, fabrics, and upholstery. In particular, PBDEs are widely found in the plastic parts of electrical and electronic equipment (EEE) and consequently need to be considered in WEEE management. 

Being synthetic compounds, PBDEs are found marketed in different congeners mixtures, each of which has an exact composition depending on the manufacturer. Table 2 presents average compositions of “penta”, “octa”, and “deca” mixtures (based on [25]).

Tetrabromobisphenol A (TBBPA) is another BFR commonly used in EEE plastics and is currently the most extensively applied BFR globally, comprising approximately 60% of the market [26]. The Globally Harmonized System (GHS) classifies TBPPA as H410: very toxic to aquatic life with long lasting effects. In June 2017, the European Union amended Annex III of Directive 2008/98/EC (Waste Framework Directive), regarding classification of waste as hazardous, setting concentration thresholds for waste containing substances declared as HP14 “Ecotoxic” by the GHS. According to this amendment, WEEE containing substances classified as H410, such as TBBPA, in concentrations higher than 2500 mg/kg are to be labeled as hazardous and their recycling is forbidden [27,28].

### 2.2. BFRs: Use and Production—Regulatory Scope

Some brominated flame retardants, such as hexabromobiphenyl (HBB), HBCDD, and PBDEs, are known to be toxic and persistent once released into the environment. Globally, there is concern about the potential environmental pollution that BFRs (especially PBDEs) from discarded or recycled WEEE can cause. This has led different countries to take measures to reduce the use of these chemicals in electronic products. Consequently, they have been classified as persistent organic pollutants (POPs) by the Stockholm Convention. This convention is what is known as a multilateral environmental agreement (MEA) overseen by United Nations Environmental Programme (UNEP), between 184 parties, of which, to date, 152 have ratified it [29]. This convention establishes that signatory countries must take administrative and legislative measures for its implementation in order to collaborate locally and globally in mitigating the impacts associated with POPs. The target chemicals included by the convention are listed in three annexes: Annex A (Elimination), Annex B (Restriction), and Annex C (Unintentional production). For chemicals listed in Annex A, parties must take measures to eliminate their use and production. For those listed in Annex B, parties must take actions to restrict their use and production and lastly, for those listed in Annex C, parties must take measures to reduce their unintentional release. 

There are currently five groups of BFRs listed in Annex A (Elimination) of the Stockholm Convention: HBB, HBCDD, commercial mixture c-decaBDE (decabromodiphenyl ether), and tetra-, penta-, hexa- and hepta-bromodiphenyl ethers. The convention mandates that waste items that contain concentrations of these BFRs higher than those defined as acceptable, must be disposed of by processes that ensure their destruction or irreversible transformation [30]. 

In some cases, certain parties are allowed to derogate from the established limits, for example, until 2030, penta- and octa-BDE are allowed to be present in waste materials sent for recycling. However, in these cases the restrictions for the production and use of these BFRs are clearly defined in the corresponding guidelines. 

While the definition of limits for the production, use, and presence in waste sent for recycling of the POPs listed in the convention helps the removal of POP-BFRs from the environment, one of the most important challenges currently faced is the substitution of these compounds by other FRs, resulting in the legislation established by the signatory countries being only partially effective. 

Other MEAs promoted and developed by UNEP include the Basel Convention on the Control of Transboundary Movement of Wastes and their Disposal, and the Rotterdam Convention on Certain Hazardous Chemicals and Pesticides. These conventions encourage international cooperation between signatory countries, which include developed and non-developed countries, to achieve certain goals or to exchange technology, resources, and knowledge.

#### 2.2.1. The European Union and the United Kingdom

In the European Union the requirements of the convention are enforced in both Directive 2011/65/EU (RoHS Directive) and Directive EU 2019/1021/EU (POPs Regulation). The first restricts the use of certain hazardous substances in electrical and electronic equipment while the latter establishes concentration limits for POPs found in waste. The two directives operate in tandem and between them define concentration limits for certain hazardous substances, including BFRs, in consumer articles and waste. 

Following life cycle logic, firstly, the RoHS Directive defines the UTCs (Unintentional Trace Contaminants) concentration limits for consumer articles entering the market including both new articles and articles that have been manufactured from recycled materials. Once the product is disposed of to be classified as waste, concentration limits are defined by Directive 2019/1021/EU, stating that in cases where these limits are exceeded, the materials may not be disposed of by conventional means, such as in landfills or recycling, but must be treated by methods that ensure their destruction (e.g., incineration for energy recovery). If these POP-containing wastes are treated in such a way that the hazardous substances are separated from the article, then it is permissible for such articles to be disposed of by conventional means. 

The concentration limit for EEE in the European Union is set at 0.1% by weight for the sum of all PBDE congeners found in the relevant article placed on the market. The POPs regulation states that the concentration limit for the sum of tetra-BDE, penta-BDE, hexa-BDE, hepta-BDE and deca-BDE is 0.1% by weight (1000 mg/kg). Any disposed item exceeding this limit is considered hazardous waste and is required to be either destroyed or irreversibly transformed. A limit of 50 mg/kg is set for HBB, commonly used as FR in ABS plastics, and 1000 mg/kg for HBCDD, commonly found in XPS and EPS (insulation materials).

In addition, a limit of 10,000 mg/kg is defined for wastes from thermal/incineration processes, metallurgical processes, construction and demolition, or industrial processes. 

In the UK, administrative and legal requirements relating to the convention are enforced by the POPs Regulations 2007, which regulated the management of WEEE containing levels of POPs above set limits [31]. As this regulation implements Directive 2019/1021/EU, said limits are the same as those applicable in the European Union.

#### 2.2.2. The Americas: South and North

The country with the highest WEEE generation volumes in South America is Brazil, a signatory of the Stockholm Convention. This country is currently benefiting from several exemptions for the application of PBDEs. For instance, the use of tetra-, penta-, hexa- and hepta-BDE is included as an exception in accordance with the provisions of Part IV of Annex A of the Stockholm Convention, thus, articles containing these compounds may still be marketed and/or recycled in Brazil [32]. To date, there are no legal requirements in place in Brazil regulating the application, import, or export of these POPs, and neither is the management of waste containing them. 

As for North America, the United States of America stands out as the largest WEEE generator of the region; however, there are currently no federal restrictions related to BFRs. There are, nevertheless, state restrictions implemented in 13 states [33].

#### 2.2.3. Asia

China, Japan, and Indonesia, three of the major EEE producers in Asia, have adopted all the annexes relating to BFR-POPs in the Stockholm Convention. While Indonesia has not put in place any national regulations or policies limiting POP concentrations in goods, both Japan and China have established strategies aligned with the convention requirements to cut back on the use of these chemicals. 

In China, the government has established various regulations that promote and standardize requirements for proper recycling processes of WEEE, including defining where they should be located and what pollution control measures should be put in place, such as dust removal or negative pressure workbenches to prevent their dispersion [34].

As for India, the other great EEE producer in this region, it has ratified only the first 12 listed POPs. Nevertheless, this country has banned use and trade of certain PBDEs (penta- and octa-), HBB, and HBCDD. Additionally, a concentration limit of 0.1% wt is legally enforced on certain EEE [33].

#### 2.2.4. Africa and Oceania

To date, 53 countries in Africa are signatories to the Stockholm Convention, many of which have limited capacity, both technical and economical, to address and battle the health and environmental issues related to POP-BFRs. No policies or regulations are currently in place in any of the signatory countries. In the case of Oceania, all 14 countries are signatories to the convention.

#### 2.2.5. Limitations

The increase in the use of NBFRs may be perceived as a limitation to the effectiveness of the Stockholm Convention and the legislation established thereunder. In order to meet flammability standards, new chemicals not currently included in the relevant legislation will enter the market. These replacements may themselves be classified as hazardous in the future due to the similarity of their chemical characteristics to those of the currently banned compounds. 

The WEEE and RoHS Directives have triggered the substitution of BFRs by other alternative Br-free flame retardants such as aluminum, magnesium, or phosphorus-based additives. Currently no legislation regulates the replacement of compounds, which in addition to creating a loop in the presence of hazardous compounds in consumer articles and later in waste, directly affects the development of detection and removal methods for POP-BRFs, as these will need to be effective for both the legacy and novel BFRs present.

#### 2.2.6. WEEE Management

There is currently no global agreement on WEEE management policies, however, in top generating countries/regions, WEEE is regulated according to legislation summarized in Appendix A: WEEE LEGISLATION WORLDWIDE.

## 3. Determination of the BFR Concentration in WEEE Plastics

The use of currently restricted BFRs has been extensive in a wide variety of applications in the past. Consequently, there is a growing inventory of materials containing POPs-BFRs that have been or will be disposed of as waste, and therefore require the development of systems that allow for their proper and sustainable management. 

Agreements, such as the Stockholm Convention, address this issue; however, although treatment and concentration limits are defined, the available technology for effectively screening consumer or waste articles in many cases is not adequate due to the precision required for their correct sorting. 

Techniques such as GC/MS or LC/MS are very precise and specific, but nevertheless require extensive preparation of samples, making them slow and expensive, so they would not be operable on an industrial scale. On the other hand, there are cheaper and faster technologies, such as handheld X-ray fluorescence (h-XRF) or Fourier transform infrared spectroscopy (FTIR). Neither of these techniques allow the determination of the concentration of the different species of BFRs found in the sample; however, they have shown potential for the screening of materials based on total bromine concentration with a high degree of certainty. In addition, h-XRF currently has limited potential for wide application due to the sources of error associated to the lack of commercially available calibration materials for different polymers and Br concentration ranges. 

As industrial scale recycling of WEEE plastics is usually done at a rate of 1 ton per hour of plastic processed, h-XRF would not be able to comply with the screening requirements of such processes. For large-scale use, both technologies raise considerations that must be examined: FTIR in an online system does not require many operators for its use, but is more expensive than h-XRF whilst h-XRF will probably require more than one unit and a number of operators trained in the use of the instrument to provide a local system to physically analyze the articles.

Improvements are continuously being made to the technologies and methods used for screening in order to increase their performance. The method most commonly used at the industrial level to separate BFR-rich from BFR-poor fractions of WEEE plastics is the sink/float method. These density separation processes are applied, whereby high-density fractions containing dense plastics and plastics containing various additives are separated from the low-density fraction with low percentages of additives. The latter fraction is subjected to further separation processes, e.g., electrostatic separation, through which the production of purer fractions of different polymers, e.g., ABS, PS, PP, and PE, is achieved. It is estimated that in the high-density fraction, more than 95% of the BFRs are contained together with other additives and intrinsically heavy plastics, such as PET, PC, PVC, etc. [3].

In a recent study, Strobl et al. [35] assessed the efficiency of a density-based process (sink/float) to separate plastics from cathode ray tube (CRT) TVs and liquid crystal display (LCD) TVs into the two BFR fractions using X-ray fluorescence (XRF) analysis. Results showed that, at the lab scale, an effective separation of the halogenated (Br-rich) from the non-halogenated (Br-poor) fractions was possible by density separation. Additionally, it was determined that the halogenated fraction of CRT TVs plastics corresponded to approximately 70% while a different trend was observed for LCD TVs with halogenated fractions varying between 47 and 72%.

In order to be recycled, plastics need to be segregated into classes according to the BFRs they contain and their concentrations, however, current plastic sorting technologies such as X-Ray Transmission (XRT) or XRF are not capable of distinguishing between, and therefore quantifying, the different types of BFRs present to determine compliance with regulation. At most, these methods allow the determination of total Br concentration. 

To address this challenge, in the EU the European Commission requested CENELEC to develop a standard setting requirement for collection, transport and treatment of WEEE. The result was the EN 50625 standard, which is currently legally binding in Belgium, Ireland, Lithuania, the Netherlands, and France [3]. In the UK, the same requirements are established in Standard BS EN 50625, but are not legally binding. In TS 50625-3-1 (and PS CLC/TS 50625-3-1:2015 in the UK) it is established that, for plastics fractions from CRT screens, flat panel display (FPD) screens, and small appliances, the threshold to segregate BFR-rich fractions is set at 2000 ppm of total Br. 

Nevertheless, the EN 50625 concentration limit was statistically determined and therefore might be inaccurate. Consequently, other sorting technologies are being evaluated that allow for a segregation of plastics both by BFR concentration and polymer type. These include methods such as Fourier transform infrared spectroscopy (FTIR), direct analysis in real time–high resolution mass spectrometry (DART–HRMS), Raman, laser-induced breakdown spectroscopy (LIBS), multi-wavelength spectroscopy coupled with multivariate statistical analysis (MWS-MVSA), and, more recently, hyperspectral imaging (HSI). Table 3 includes a brief description of each of these methods.

### The Challenge of WEEE Classification according to Br Content

In several recent studies carried out in Australia [40], Austria [41], the Czech Republic [13], and the UK [42,43,44], authors agree that the classification of WEEE plastics by defined EEE categories is unlikely to be feasible. Thus, it was suggested that the best approach would be to separate and short the plastics in order to avoid misclassifying BFR-free plastic parts as BFR-rich, and vice versa. Contrary to these conclusions, in the UK, the Environmental Agency published a guideline based on Keeley et al. [45], where WEEE are classified as POPs/hazardous waste according to their EEE categories. 

In Europe, the current capacity to separate and dispose of BFR plastics collected by official means is 215 kt/year (92% of the total WEEE/BFR plastics generated annually); however, it is estimated that around 120 kt/year are incorrectly managed in sub-standard recycling facilities, exported, or disposed of in bins with other general waste. Thus, the low volumes of treated WEEE/BFR plastic and, in contrast, the high volumes that are potentially released into the environment, are not linked to the installed processing capacity, but rather to inadequate sorting of WEEE by consumers and sub-standard recycling practices. 

A common practice in the recycling industry is to shred mixed WEEE plastics, which might be then pelletized and later separated by density in a sink/float process. To assess the possibility of using h-XRF for Br determination and plastic sorting, Stubbings et al. [14], studied extruded polymer pellets which were sorted by density separation into three fractions: light/medium/high. Each fraction was characterized for 28 legacy and novel BFRs; handheld XRF was used to determine total bromine concentration in 120 individual chips of various polymer types. A very important observation was made as Br concentration within different density classes was determined to be linked to the heterogeneity of the process’s material. This means that each pellet consisted of melted plastic from many sources, thus the concentration of BFRs present in a pellet is an integration of the BFRs present in the individual plastic parts of each individual original item. Consequentially, plastics with low BFR concentrations (compliant with regulation) are mixed with plastics with high BFR concentrations resulting in pellets that do not comply with POP concentration limits. Therefore, the plastics are classified as POP waste, resulting in the loss of recyclable materials. 

Considering the above, it can be concluded that, while the classification of WEEE by EEE categories might not be the most effective approach for a proper classification of plastics according to their BFRs content, resorting to their classification from mixed shredded and extruded WEEE plastics needs careful design for it to be accurate. This highlights the importance of sorting plastics at a primary stage, prior to or after shredding but before palletization to increase the volumes of plastics plausible of being sent for recycling according to the POP concentration limits defined in the relevant regulation.

In addition to the efficiency of the separation method and the quality of the separated material, costs are one of the most important factors in determining the viability of WEEE plastic recycling operations. The costs of this process have different inputs, including those related to sorting, processing, and disposal. As a counterpart, there are profits resulting from the sale of the materials, which increase with the efficiency of the overall treatment process. 

As previously discussed, in order to comply with current regulations, it is mandatory to separate the regulated BFRs plastics, which entails separation and disposal costs for the fraction that cannot be sent for recycling. However, separation processes are key to recovering both homogeneous polymer fractions and those with low additive concentrations, therefore the investment and costs associated with separation processes cannot be allocated solely to the separation of BFR plastics. Likewise, the costs associated with the disposal of the high-density polymer fraction (containing high concentrations of BFRs) also cannot be attributed solely to the BFR content, as this fraction is already not suitable for recycling. On the contrary, if the BFRs present are POPs, then there will be additional costs as the material must be sorted and therefore treated as hazardous. 

Furthermore, to add value to the material sent for recycling, WEEE plastics should not only be sorted into BFR/non-BFR fractions but also by polymer type and colour (black/clear). Depending on what methods are applied and in what order, the efficiency of the process varies and so do the costs. The challenge is to design a process producing high quality material, compliant with regulations, that can be sold at higher prices with costs below revenue. 

## 4. BFRs Distribution in WEEE Plastics

Several studies have been carried out over the past decade to determine the BFR concentration in WEEE plastics. For instance, in a study by Hennebert and Filella [24], the concentration of total Br and specific BFRs were determined in EEE and WEEE samples taken between 2009–2013 (EEE) and 2014–2015 (WEEE), for their regulatory classification. It was determined that the most concentrated BFRs found in WEEE were deca-BDE and TBBPA (3000 mg/kg and 8000 mg/kg, respectively), with 86% of total Br found in small household appliances (older waste) and 30–50% of total Br found in flat screens (younger waste) corresponding to regulated brominated substances. 

An assessment of Irish waste polymers, carried out by Drage et al. [12], considered four waste streams that were defined as most likely to contain BFRs, including WEEE. A total of 239 WEEE samples were analysed to determine their content of PBDEs (penta- and octa-), HBCDD and BDE-209 (decabromodiphenyl ether) in order to establish what percentage of the samples exceeded the LPCLs (low POP concentration limits) established by regulations. In addition, information on the volumes of waste annually generated in Ireland was collected. To estimate the volumes of waste that would annually require treatment for the removal of POPs-BFRs or treatment that ensures their destruction, the volumes of generated waste were paired with that obtained from the BFR content analyses. It was concluded that approximately 2200 tons/year of waste generated in Ireland contained levels of POPs-BFRs higher than the LPCLs set out in the regulation, with 13% being WEEE. It should be noted that at the time of the study BDE-209 had not been included in the POPs Directive (Directive 2019/1021) and a threshold value of 1000 mg/kg BDE-209 was taken as set out in the Stockholm Convention. Currently BDE-209 is included within the limit of the sum of PBDEs (together with tetra-, penta-, hexa- and hepta-) that cannot exceed 1000 mg/kg, so it is likely that the percentage of WEEE that exceeds the LPCL and cannot be directly recycled is currently higher.

A later study by Jandric et al. [16] evaluated six types of WEEE devices which were manually dismantled, and their parts analysed to determine the different types of polymers, as well as total Br concentration. Results on Br concentrations were presented as a function of the device type, the polymer type and the year of manufacture. It was determined that 35% of the samples contained Br concentrations exceeding RoHS limits (0.1% of PBBs and PBDEs). It is worth noting that as a limitation to this study a conversion factor was determined by Aldrian et al. [41] to convert total Br concentration into PBDE or PBB concentrations. In the latter, other BFR types (TBBPA, HBCD, compound mixtures) were not considered. 

In the same year, Stubbings et al. [14] published an assessment of BFRs in small mixed WEEE where pellets of extruded mixed polymers were analysed to determine the presence of legacy and NBFRs in the different types of polymers. The aim of the study was to determine which are the polymer types that may not comply with Directive 1021/2019 in the EU and POPs regulation in the UK. It was determined that BDE-209 and TBBPA were the main BFRs found, and they were present in all samples, with concentrations ranging between 68–37,000 mg/kg and 17–120,000 mg/kg, respectively. It is relevant to remark that 22% of the samples were misclassified as POP waste. This result was higher than that obtained by the study carried out in Ireland, [12]. It is suggested that the error might be related to the lack of calibration reference material for the higher concentration range.

A summary of the studies presented above is included in Table 4.

## 5. WEEE Plastics Treatment: The State of the Art

Efforts are being made to address the issue of BFRs presence in WEEE plastics to increase the recyclability volumes and economic benefits. There appear to be two different approaches to researching and developing solutions to this challenge. On the one hand, the focus is on replacing legacy BFRs with novel FRs that are not currently restricted, while on the other hand, work is being done on the development of processes for the efficient extraction and recovery of BFRs and hazardous synergists, and on the recycling of clean plastic.

The approach by FR substitution has raised concerns in the plastics manufacturing and recycling industries as these new additives, even though complying with current legislation, may affect the quality of the material by deteriorating the polymer structure and could potentially result in the plastic no longer being recyclable [3]. 

As for WEEE plastic cleaning and additives recovery, there are three projects underway in Europe: CREAToR (1 June 2019–30 November 2022), Plast2Bcleaned (1 June 2019–23 May 2023) and NON-TOX (1 June 2019–31 May 2022). The three projects are funded by the European Union’s Horizon 2020 research and innovation programme and aimed at removing hazardous flame retardants from plastic waste. In the CREAToR project, materials targeted are ABS/PC, ABS, and HIPS plastics and treatment is to be performed by wet material handling of particles sized between 40 to 60 mm. Targeted elements are heavy metals, including Cd, Pb and Cr, BFRs and chlorinated flame retardants (CFR). The aim of this project is to reduce dependency on petroleum sources, generate job opportunities in the sector, and turn waste into non-hazardous resources. Challenges identified include seeing black plastics due to their low reflectance in the NIR region (being these the method most commonly used at industrial scale for polymer sorting), development of automated sorting processes and achieving a good ratio of purity/efficiency. Attending to these challenges, solutions proposed include using density baths to separate heavy from light plastic fractions, polymer sorting using multi-wavelength infrared radiation (MWIR) and the use of LIBS combining polymer and element detection of Br, Cl, Pb, Cd, and Cr [46].

The objective of the PLAST2bCLEANED project is focused on HIPS and ABS plastics. Its aim is to develop a mechanical process using LIBS and Raman spectrometry that will lead to identifying several polymers of any color, which contain different additives like BFRs or pigments to separate HIPS and ABS fractions. BFRs and Sb_2_O_3_ will be recovered from separated plastics using superheated solvents. This project is looking at closing the polymer-Br-Sb_2_O_3_ loop by integrating processes and scaling them up, aiming to achieve 80% recovery yields and 8% increase in recycling rates while eliminating 40% of CO_2_ emissions

In the NON-TOX project, an automated highspeed sensor (AHS) is used for sorting plastics (80% efficiency has been achieved in classifying BFRs). The main recycling fraction is the treated by two processes:

1. Subjected to Creasolv^®^ and Extruclean^®^ technologies for the extraction additives, while maintaining the polymer’s properties. Three different streams are obtained with this process: targeted polymers with halogen concentrations below 1000 ppm, a halogen concentrate, and a mix of all the other plastics;
2. Subjected to thermochemical conversion where, at appropriate temperatures, halogenated polymers release the halogens contained in them.

The expected impact of this project is to increase the EU recycling capacity by 74%, reaching 2.18 Mt of plastics processed per year, while creating jobs and reducing emissions. 

A positive outcome of these projects would mean a significant improvement for the WEEE plastics recycling industry in the EU, increasing processing capacity, job opportunities, and reducing the associated negative environmental impacts. 

## 6. BFRs Existence in Consumer Product Plastics

Several studies have determined the presence of BFRs in consumer products (CPs) and in other products, through which humans may be exposed such as toys and jewellery. The presence of these compounds in CPs plastics is explained because of improper handling of WEEE plastics that are mixed with virgin polymers. In Europe this constitutes a violation of the regulation (Regulation EU 10/2011). However, the use of recycled polymer for the manufacture of CPs is allowed in the European market but only under strict conditions that include a careful and efficient separation of the materials to be recycled. Particularly in the case of black plastic, the plastic sorting processes currently implemented in the industry are not able to separate them as most of them are based on IR.

Consumer products containing BFRs, particularly items that come into contact with food (food contact articles—FCAs) or small toys that children can put in their mouths, compromise consumer safety as these plastics directly affect the quality of the item and can release certain levels of different chemicals through migration, thereby exposing the consumer. A number of studies have determined farther sources of exposure to these hazardous chemicals. For instance, in a study by Yu et al. [15], the content of PBDEs and TBBPA in household waste was quantified to identify the sources and trends of these compounds and understand the potential for migration of PBDEs and TBBPA into consumer products via plastic recycling processes. It is reported that results support conclusions from previous studies about the importance of separating WEEE plastics containing BFRs from other plastics that can be recycled to be safely used in consumer goods. Furthermore, disposing of WEEE plastics in the municipal waste stream in landfills or through incineration, constitute a means for BFRs, Sb, Cd, and Pb to be released into the environment. 

Based on a previous study by the same authors, the work in [13] investigated the potential contamination with WEEE stream polymers of polymeric FCAs in more detail by using different techniques to prove the illegal use of recycled WEEE plastic in black plastic. According to European Commission Regulation (EC) No. 10/2011, this is an illegal practice within the European Union. The study confirms the indication that black polymer FCAs manufactured using recycled WEEE plastic circulate in the European market. Two years later, the same author published in [47] a study linked to that of 2015, aimed at proposing a method based on levels of significance for an efficient in situ assessment of the presence of WEEE plastics in FCA plastics by determining Br and Sb (level 1), BFRs (level 2) and WEEE-related elements and additionally the purity of the polymers (level 3). 

Another study by Turner and Filella [44] assesses the ubiquity of Br in consumer products’ plastics including electronic, electrical, and non-electronic items. Total Br concentration was determined by XRF spectrometry as an indicator of BFRs presence in these plastics. It was concluded that high concentrations of Br are found in consumer products which could potentially results in human exposure to BFRs (as well as Sb and Pb usually found associated with Br in plastic items). Determining the presence of Br and Sb in plastics of items that do not require flame retardants suggests that the plastic components of the old items were and/or are being recycled into consumer products.

Consistent with previous studies, Turner and Filella found that most Br-contaminated consumer products are black in colour, even though BFRs are added to plastics in electronic goods in a range of mostly neutral colors. The authors conclude that this is linked to the practical difficulties and costs associated with recycling plastics pigmented with black carbon. Particularly, black plastics cannot be sorted according to polymer type by optical means as the black pigment absorbs infrared radiation. This results in a limited recyclability of black plastic in contrast to the high demand for black plastic items, which may lead to manufacturers using (intentionally or unintentionally) black plastic from WEEE containing BFRs, Sb, and Pb as an alternative source of this material for the manufacture of different consumer products. 

Further research on the issue of WEEE black plastic recycling for the manufacturing of consumer products was carried out by Turner, who in 2018 [17], published a literature review in which it is identified that, while the manufacture of plastics in general already generates environmental and health impacts, the production of black plastics in particular has greater and more serious risks associated with it. It was then stated that such impacts are a direct consequence of the low separation efficiency of these plastics in the industry, related to the sorting and separation methods that are currently in place. In the same review, the linearity or circularity of the WEEE black plastics economy is assessed. It is determined that black plastics are embedded in a quasi-circular economy model, in which the material is inefficiently sorted while being highly demanded by the manufacturing industry to partially cover black plastic requirements for the production of consumer materials. As the plastic is not properly sorted, the material that is recycled does not meet the requirements regarding the content of BFRs and these hazardous additives end up being present in consumer goods, which significantly increases the exposure of humans to these compounds and thus affect their health.

Another source of exposure to BFRs through CP plastics are toys. To assess this, Fatunsin et al. [48] carried out a study in the UK, selecting 23 plastic toys components dating between 1997 and 2017. The aim was to confirm previous evidence of WEEE plastic recycling into consumer products and children potential exposure to BFRs (including PBDEs, TBBPA and HBCDD) by accidental ingestion. Results of the study confirmed the presence of BFRs in 20 of the 23 analyzed samples, which, as components of consumer products, are not included in the regulations for flame retardants. This evidence adds to previous studies indicating that the presence of BFRs in consumer product plastics must be linked to the recycling of WEEE plastics. On the other hand, the evaluation of the exposure of children to BFRs through accidental ingestion indicates that, in some cases, the exposure is considerable, being for some individuals the most significant route of exposure to BFRs. These observations disclose the need for stricter controls in the recycling industry to ensure the removal of BFRs from WEEE plastic streams sent for recycling, as well as a probable lack of efficiency in the processes currently applied for the separation of plastics containing BFRs. Furthermore, the fact that children can potentially be exposed to these hazardous compounds through their contact with toys highlights the importance of further controls, research, and development in the processes WEEE plastics recycling. 

Despite the increase in the use of NBFRs there is currently not enough knowledge on the potential cumulative effects of any NBFRs and no analytical methods have been determined to measure these compounds, with a research gap so large that according to a review by Zuiderveen et al. [25], 28 out of the 63 NBFRs studied are not even included in monitoring programs or studies exposing the need for further research on the toxicity and fate of NBFRs, for which their presence in consumer goods has already been determined. 

## 7. WEEE Recycling as a Source of Exposure to BFRs: Health and Environmental Hazards

In most countries, WEEE generated at the household level is managed either through disposal in waste bins, through informal collection by private companies or governmental organizations, or through collection outside the formal system by informal waste management companies. WEEE collected through the formal system is generally treated through processes that ensure safe treatment for the environment and health. On the other hand, those who manage these wastes illegally mostly process them under basic technological conditions and without measures to prevent emissions of hazardous compounds such as BFRs. The persistence over time of these informal activities combined with the persistence of BFRs in the environment increases the level and risk of exposure of both the workers involved and those living nearby, and consequently represents a potential health hazard. In the developing world, which is the largest generator and recycler of WEEE, a vast majority of such recycling units are in the informal sectors that usually lack advanced technology for WEEE recycling and are not able to take effective measures for mitigating environmental pollution associated with inefficient WEEE recycling. 

Particularly, in China and developing countries where informal recycling of WEEE is common, due to the high degree of application of BFRs in EEE plastics, and as a consequence of dismantling, acid treatment, and open burning of WEEE, these hazardous compounds are being released uncontrollably into the environment. Concentrations of TBBPA and PBDEs have been detected in vegetables, rice plants, and other wetland plantations, indicating that these BFRs may enter the food chain through accumulation in plants and soil. In addition, their presence has been detected in molluscs, birds, and humans (blood, adipose tissue, and breast milk). This highlights the importance of addressing the management of WEEE plastics to prevent these hazardous compounds from affecting the environment and health.

Even in an organized sector, WEEE recycling poses several environmental and occupational health hazards which also partly stem from the fact that WEEE recycling technologies have not kept pace with the technological advancements in the field of production of electronic and electrical equipment. 

Recently, several studies were carried out to assess the sources of exposure to BFRs and related impacts to the environment, animals, and humans, with a particular focus on people working at either a WEEE recycling site or residing in nearby areas. The first reported flame retardant exposure study was carried out at four WEEE recycling sites in Finland between 2008 and 2009 by Rosenberg et al. [49]. For the assessment, air concentrations of five brominated flame retardants were measured in breathing areas. It was observed that the most abundant FRs in the personal air samples were PBDEs, TBBPA and DBDPE, and it was found that in two of the four study sites, emission control measures (such as improvements in ventilation and changes in maintenance and cleaning habits) had positive results in decreasing the levels of RFs present in the air. 

In 2020, Cai et al. [50] published a systematic review addressing the current status of human exposure to PBDEs in WEEE recycling areas in different countries and identifying their effects. The review includes studies from different geographic areas in various countries around the world, such as China, India, Vietnam, Thailand, Nigeria, Ghana, Canada and the United States. It was then determined that risks of long-term exposure of humans to PBDEs due to improper handling and recycling of WEEE have existed in the past and that particularly in China, exposure levels in informal WEEE management areas are at least an order of magnitude higher than in other areas. 

An important conclusion was drawn relative to the route of exposure to PBDEs, where ingestion through food was considered the most relevant source, while air inhalation and dust ingestion are also important mainly in workers in the sector. Furthermore, evidence show that those residing in WEEE recycling areas for longer periods of time, would have been exposed to considerable levels, and children are likely to face higher exposure due to a wider variety of exposure pathways including placental metastasis, ingestion through breast milk, behaviors such as ingestion of soil or putting objects in the mouth, among others. WEEE recycling workers are also in the high-risk group, as they are exposed to higher levels of PBDEs and for longer periods of time, which could be determined through studies of hair, serum, and some tissues. In sum, it is concluded that exposure to PBDEs is higher in WEEE recycling areas.

In a previous study by Die et al. [34], similar observations were made and more details about sources of exposure were determined. The study aimed to determine the concentration of PBDEs in air, soil dust and fly ash samples obtained from a bag-type dust collector in different WEEE recycling plants, and to estimate the daily intake of PBDEs by workers through inhalation or ingestion of dust. The results showed a high concentration of PBDEs in all three monitored matrices in TV and plastic WEEE recycling plants. It was also found that the concentration of PBDEs in the air decreased with increasing efficiency of the dust removal equipment installed in the different facilities. However, the exposure of workers to PBDEs was found to be within safe levels, and in some cases up to two orders of magnitude lower than those defined by US EPA as reference doses. These results highlight the need for strict pollution control measures to be put in place in WEEE recycling plants, mainly dust collectors to help reduce worker exposure to hazardous compounds, such as PBDEs.

It was also found that daily exposure of PBDE of workers from these plants was lower than that found for workers of other recycling facilities. Additionally, it was determined that the ingestion of dust was the main route of exposure for workers doing manual recycling, while inhalation was the main source for workers in charge of waste transportation. 

Overall, it was concluded that the emissions of PBDEs and the associated risks, are lower in modern formal WEEE recycling plants where effective pollution control measures are in place. Nevertheless, it was highlighted that further research is understood to be necessary to determine how effective these controls are in plastic crushing areas where dust is found to have the highest PBDEs concentrations. 

This was in line with that exposed in a more recent review by Ma et al. [51], who determined that the three main routes of external exposure to halogenated FRs in humans at informal WEEE handling sites are ingestion of dust and food, inhalation, and dermal absorption. However, there is currently little research on exposure through dermal absorption. It also agrees with the conclusion that workers in informal WEEE recycling plants, children, infants, and fetuses, are subject to the highest exposure compared to adults who reside in the plant area but do not work in this activity. In this review further observations were made as it was noted that some studies report on existing differences in internal exposure to BFRs according to gender, with higher concentrations of BFRs found in serum and hair of females compared to those found in males. However, the cause of this is still unclear. 

As hair is one of the non-invasive matrices for the study of biomarkers in humans, as well as being easy to sample and inexpensive to store and transport, several studies are based on the study of hair samples and have shown that hair samples can be used to differentiate between study subjects and exposure levels. It is important to note that particularly in hair, the contaminant can enter via internal (through the blood) or external (through exposure to dust, air, or chemicals applied to the hair) routes. In a study carried out in 2015 by Qiao et al. [52], hair samples from 31 female employees of WEEE recycling facilities were analyzed to determine the impact on the body of their exposure to legacy and novel FRs, and to analyze the accumulation of these compounds in hair by identifying which factors promote or prevent such accumulation. The results of the hair analysis indicated a mean concentration of the sum of PBDEs of 154 ng/g, 92.8% of which corresponded to BDE-209. Additionally, samples were taken from the hands of 10 workers during their working hours and 10 samples of dust from the floor inside the facility in order to characterize the external sources of exposure. However, the floor dust samples represent a single external exposure pathway, while samples taken from hands reflect exposure through contact with dust and WEEE. Although high concentrations of contaminants were determined in the wipes, a relationship between the contaminants determined in the wipes and the hair could not be determined. 

In a review by Gravel et al. [11], peer-reviewed publications of quantitative air, dust, and biological fluid studies assessing the occupational exposure of formal workers from different industries to organic compounds, including BFRs were critically collected and compiled. Evidence showed the most studied compounds were PBDEs and NBFRs, while the most studied sites were offices and WEEE recycling facilities, the latter reporting the highest exposures. Nevertheless, most of the studies concerning the health effects of FRs have been carried out in children, animals or in vitro studies, so, as the effects of exposure in adults have not been studied in depth, the evidence is scarce and no OELs (occupational exposure limits) have been defined. A study by Shen et al. [53] assesses the gap in toxicity data of NBFRs discussing the neuro (endocrine) toxic effect of NBFRs both in vitro and in vivo. Data from this article indicate that, particularly during early neurodevelopment, exposure to NBFRs could cause unwanted neurobehavior with potential to damage the neuroendocrine system including affects to thyroid/sex hormone levels. Current data and published studies on the health effects of NBFRs suggest that it is imperative to further study their impact on behavior and brain function, as well as the changes in structure that result from exposure to NBFRs, as well as the neurotoxicity of their metabolites and related breakdown products [54]. A similar observation was made by Li et al. [55], who also identified a research gap on the evaluation of the effects of NBFRs to humans as studies are mainly in vitro and in vivo animal studies, where their health effects were studied through acute toxicity, endocrine disrupting activity, reproductive, and developmental toxicity, among others. It was determined that airborne particles are the main carriers of FRs and therefore contribute significantly to their inhalation. On the other hand, it was found that dermal absorption of FR particles also contributes considerably to the overall intake of these compounds. This is in line with previous studies of legacy BFR exposure of WEEE recycling workers. However, in most of the cases evaluated, the FR intake doses were estimated to be substantially below the reference values. In a recent study carried out by Ling et al. [56], the contamination of water and sediments by legacy and novel BFRs was evaluated at two of the world’s largest e-waste dismantling sites located in the Chinese city of Taizhou. According to the results it was found that the concentration of BFRs and NBFRs in the water of both sites were comparable and at the upper end of the global range, while in the sediments there were differences with high concentrations at one site and relatively low concentrations at the other. It could, therefore, be proven that the WEEE dismantling activity has posed an unacceptable risk to animals living both in the water and sediments surrounding the sites. This shows that even in developed countries, such as China, it is necessary to reinforce the legislation on BFRs in order to define risk control standards for the environment, including water, sediments and other matrices that may be affected and have not yet been studied. At the same time, the high levels of BFRs and NBFRs concentration in the surrounding areas show that measures should be taken to optimize the e-waste dismantling process carried out there, seeking to eliminate the informal management of these wastes. It is also of utmost importance to continue studying the risk to the health of the workers as well as the population in the area.

It should be noted that most of the studies of human exposure to BFRs have been carried out in China and considering this country banned the import of WEEE in 2018, it is likely that these wastes are being directed to other countries or regions, which will have a corresponding environmental and health impact if they are improperly treated. It is therefore important that studies are carried out on the informal treatment of WEEE in low- and middle-income countries.

## 8. Knowledge and Research Gaps

The issue of BFRs in WEEE plastics has been address by different projects and authors in the past decade. As previously discussed, according to the Stockholm Convention, a number of PBDEs and HBBC are identified as POPs and are therefore restricted. However, there are currently alternative BFRs being used with similar chemical characteristics to those restricted which could thus be later classified as POPs. 

In addition to this gap in regulations which will likely result in larger volumes of currently unregulated BFRs being found in WEEE plastics in future years when EEE currently manufactured become waste, most studies are not considering the presence of novel BFRs in WEEE plastics arriving to misleading conclusions on the presence of these additives. Furthermore, studies in which a total Br concentration threshold is determined need to be carefully considered as in most cases not all Br components are evaluated and therefore the baseline is not accurate. For instance, a conversion factor was determined by Aldrian et al. [41] to convert total Br concentration into PBDE or PBB concentrations, however in the study other BFR types, such as TBBPA, HBCD, or compound mixtures, were not considered.

A possible solution to this could be to generate a statistically validated and comprehensive data base containing detailed information on BFR concentrations by WEEE category, device and plastic types, functionality of the components and year of production. To achieve this, the collaboration of the manufacturing industry would be key. Additionally, FRs recently incorporated to consumer products need to be studied in more depth and amplitude.

Regarding techniques and technology available for the determination of BFRs in plastics at industrial level further research into, and the development of methods to screen for hazardous chemicals in end of life materials, is of the utmost importance. This should include an assessment of the economic, environmental and health benefits of manual dismantling as pre-processing method taken to enable the production of more homogeneous and Br-free plastics fractions. In particular, the determination of the pathways of occupational exposure and the distribution of airborne FR particles in WEEE processing facilities needs to be studies in more depth. 

The restriction of BFRs in WEEE plastic respond to the fact that certain BFRs may have severe impacts on both the environment and human health and should therefore be removed from the recycling stream. However, from published literature reviews on BFRs and health, it becomes obvious that more investigations are needed to monitor potential cross-contamination when BFRs are unintentionally transferred from one product to another through recycling process. Especially, in terms of black plastic from consumer goods where further research is needed to assess the migration of the hazardous compounds introduced in consumer goods via black WEEE plastic recycling, the sources of exposure and impacts on human health.

## 9. Discussion

Directives such as RoHS and POPs, which define limits on the concentrations of BFRs accepted in products and waste respectively, have as a direct consequence that high percentages of the plastic volumes arriving at recycling facilities cannot be effectively recycled, as this requires the irreversible destruction of the BFRs contained in the material, processes that are still under development. This leaves companies with no alternative but to incinerate the plastic. As a measure to optimize the volumes of plastic that is recycled, one option would be to replace the currently used BFRs with bromine-free FRs. However, the proposal to migrate to the use of bromine-free FRs may not help to increase the percentage of WEEE plastic recycled as these would also be sorted with the high Br concentration fraction. Additionally, the effect of bromine-free FR on plastic recyclability needs to be further studied as it is still undetermined how do they affect the quality of the material regarding the purity and other characteristics of the polymer. 

An alternative approach could include development and approval of pro-active legislation that eliminates the need for using such persistent and potentially harmful chemicals in the future, and/or evaluating the flame retardancy requirements established in regulation considering the changes in materials, lifestyles, and safety measures put in place in a wider number of households and offices, such as CO_2_ and smoke detectors. 

Overall, the presence of BFRs in WEEE plastics is a matter of concern when it comes to their management and focus must be put on aiming to prevent potential negative impacts to both the environment and public health. That is why it is imperative that knowledge on the subject continues to be developed and deepened in order to provide all actors involved in WEEE management with the necessary tools to develop strategies to prevent any harmful impacts. Adequate concentration limits need to be defined that, in addition to protecting health and the environment, allow the treatment of these plastics by means that favour the circularity of the material, i.e., recycling as opposed to incineration. Currently, the industrial processes developed and in place present low levels of recyclability, which is explained by the difficulty of determining the content of restricted BFRs. It would therefore be positive to work on defining regulated limits based on total Br concentrations that favour the sorting of the material using simple and therefore less costly and more efficient methods.

In any case, it is always important to define whether the screening and characterization methods are robust enough to comply with the regulation, as well as to assess whether the defined thresholds are sufficiently conservative.

Considering the current conditions and challenges of handling WEEE plastics containing BFRs, the substitution of restricted BFRs by other compounds that potentially need to be further restricted as well (i.e., NBFRs), or the substitution by non-brominated materials that may affect the quality of the recycled material, it is inevitable to ask whether the solution can be found in another alternative. For example, could a material be produced with the same physico-chemical characteristics as petroleum-based plastics, which is also economically feasible and replaces plastic in the manufacture of EEE? Could bioplastics be considered for this purpose? And would these materials also need to contain FRs?

In the long term, the answer to the problem of BFRs and the recycling of plastics from WEEE may lie not in finding effective processes for their detection and removal, but in finding alternative materials to eliminate the need for their presence in electrical and electronic equipment. 

## Figures and Tables

**Table 1:** BFR classification.

BFR Type	Interaction with Polymer	Example
Reactive	The BFR form a chemical bond with the polymer matrix which does not cause a softening effect.	TBBPA ^1^
Additive	The BFR is blended in the polymer’s matrix. These are more prone to leach as they are known to soften the polymer.	PBDEs ^2^ HBCDD ^3^

Note: ^1^ tetrabromobisphenol A, ^2^ polybrominated diphenyl ethers, ^3^ hexabromocyclododecane.

**Table 2:** PBDEs (polybrominated diphenyl ethers) commercial mixtures composition.

Mixture	Percentage	Congeners
Penta	70%	99 (penta-BDE) 47 (tetra-BDE)
	<10%	100 (penta-BDE)
	<5%	153 and 154 (hexa-BDE)
Octa	10–12%	Hexa-BDE
	43–44%	Hepta-BDE
	31–35%	Octa-BDE
	9–11%	Nona-BDE
	0–1%	Deca-BDE
Deca	98%	Deca-BDE
	2%	Nona-BDEs

**Table 3:** Methods for sorting WEEE plastics according to their BFR contents.

Method	Characteristic Features	Reference
FTIR	Potential to provide information on BFR presence in real time. No considerable alteration of the sample is required.	[36]
DART-HRMS	Faster than common extraction/analysis. Targets molecular species: able to screen a wide number of BFRs including novel BFRs. Allows for desorption-ionization directly off the surface of the sample. Unclear if detection of BFRs is possible at relevant concentrations. Unclear if its specificity is sufficient.	[37]
RAMAN	Potential as an effective tool for rapid BFR detection in plastic when coupled with XRF.	[36]
LIBS	Elemental analysis technique. High analytical speed. Does not require excessive sample preparation. Adapts to harsh industrial environments. Intrinsic drawbacks do not allow for a thorough BFR measurement. This could be improved by combining it with XRF or inductively coupled plasma (ICP) spectrometry.	[38]
HSI	Able to measure a whole spectrum in every pixel in which the sample is divided. Linked to data analysis (chemometrics). Identification and segregation of polymer and flame retardants in plastic samples.	[39]

**Table 4:** Summary of recent studies on the determination of BFR concentration in WEEE plastics.

WEEE plastic sorting for bromine essential to enforce EU regulation [24]	**Aim**	Determine the concentrations of total Br and specific BFR
	**Sample**	EEE samples taken between 2009–2013 from a service laboratory for commercial products WEEE samples collected between 2014–2015 from processing facilities before and after shredding
	**Methodology**	ICP/OES (ISO 11885) for Sb determination, combusted oxygen in a closed system (EN 14582) and ionic chromatography for Br quantification, and GC/MS (IEC 62321-6) for determining the concentration of different BFRs
	**Results**	A 2000 ppm limit for total Br concentration can be used to classify POPs and non-POP WEEE plastic waste at laboratory scale. The contribution of BFRs to total WEEE plastic waste in weight was estimated to be about 25%
Brominated flame retardants in Irish waste polymers: Concentrations, legislative compliance, and treatment options [12]	**Aim**	Measure the concentrations of PBDEs and HBCDD Estimate the mass of waste material and associated POP-BFRs that would be removed from circulation by effective enforcement of current LPCLs (low POP concentration limits) defined in EU Directive 2019/1021
	**Sample**	538 samples of WEEE, soft furnishings, construction and demolition waste, fabrics and PUF from ELVs in Ireland. Construction and demolition (EPS/XPS) = 62 ELV fabrics and PUF = 135 Soft furnishings = 123 WEEE = 239
	**Methodology**	Quantitative analysis: GC/MS and LC-MS/MS
	**Results**	Approximately 2200 tons/year of the waste generated in Ireland contains POPs-BFR level higher than those set as LPCL in EC regulation (1000 mg/kg of PBDE excluding BDE-9209 and 1000 mg/kg of HBCDD). Articles exceeding LPCLs: 44% building insulation 41% furniture foams and fabrics 13% WEEE 1.7% end of life (EoL) vehicle waste
Investigation of the heterogeneity of bromine in plastic components as an indicator for brominated flame retardants in waste electrical and electronic equipment with regard to recyclability [16]	**Aim**	Determine the distribution of Br in plastics from WEEE
	**Sample**	882 components (369 different devices). Mixed WEEE (6 types of devices: personal computers, computer mice, keyboards, power supply units, vacuum cleaners, electrical toothbrushes)
	**Methodology**	Manual dismantling. Handheld XRF to determine total concentration of Br in different devices and different plastic components. FTIR spectroscopy to determine different polymers.
	**Results**	Br content represented by type of device, type of plastic and year of manufacture 35% of samples contain Br and 5% exceeded RoHS limit (mainly older devices). 18 different types of polymers were identified with ABS accounting for 44% of samples
Assessment of brominated flame retardants in a small mixed waste electronic and electrical equipment (WEEE) plastic recycling stream in the UK [14]	**Aim**	Identify the presence of legacy and novel BFRs in individual polymer types from WEEE, to determine which polymers may be the ones not compliant with the LPCL (low POP concentration limits) established in regulation.
	**Sample**	217 individual polymer chips
	**Methodology**	Extruded polymer pellets were sorted by density separation into 3 fractions: light/medium/high. Each fraction was characterized for 28 legacy and novel BFRs. Portable XRF was used to determine total bromine concentration in 120 individual chips of various polymer types. If the concentration of total Br in the pellet was higher than 2500 mg/kg, the pellet was further analyzed by mass spectrometry (GC-MS, LC-MS/MS). 27 of which were further analyzed by MS for BFRs. 97 chips: analyzed by MS
	**Results**	BDE-209 (68–37,000 mg/kg) and TBBP-A (17–120,000 mg/kg) were determined to be predominant and ubiquitous. 22% of chips analyzed were misclassified as POPs waste by XRF. The presence of NBFRs DBDPE and BTBPE (1,2-Bis(2,4,6-tribromophenoxy)ethane) was identified in WEEE plastics Color sorting resulted in significant reductions in the concentrations of BFRs in the clear fraction, however, white polymers still did not comply with LPCL. ABS and PS-HIPS are the major contributors to the overall BFR concentration sin the “medium” fraction. TBBP-A is known to be extensively used as an additive in HIPS and ABS for EEE. Heavy fraction: ABS, PC-ABS, PA, P, PVC, POM and PET. ABS, PC-ABS and PET were the only polymers containing BFRs in the heavy fraction

## Data Availability

No new data were generated from this study. It represents a review and synthesis of published literature.

## Appendix A. WEEE Legislation Worldwide

**Table A1:** Europe.

Generated Volume: 12 Mt Generated in 2019 [22]				
Country	WEEE Generation (kt/year)	Legislation/Regulation	Comments	Reference
European Union countries	7889	Directive 2002/96/EC of the European Parliament and of the Council of 27 January 2003 on waste electrical and electronic equipment (WEEE)—Joint declaration of the European Parliament, the Council and the Commission relating to Article 9, 2003.	Instrumented to enable environmentally sound WEEE management. Sets targets for collection and recycling for all Member States. Aimed at minimizing WEEE generation by promoting reuse, recycling, and recovery. Each Member State must devise their own strategy to reach collection and recycling targets. Mandates EEE manufacturers and importers to collect end-of-use and end-of-life EEE from consumers and properly manage them.	[57]
		Directive 2012/19/EU of the European Parliament and of the Council of 4 July 2012 on waste electrical and electronic equipment (WEEE), 2012.	Recast of Directive 2002/96/EC. Sets new collection targets. Officially replaces Directive 2002/96/EC in 2014.	
		Directive 2002/95/EC of the European Parliament and of the Council of 27 January 2003 on the restriction of the use of certain hazardous substances in electrical and electronic equipment, 2003.	Aimed at restricting the use of hazardous substances in the manufacture of EEE. These substances include: brominated fire retardants such as PBDEs (poly-brominated diphenyl ethers), POPs (persistent organic pollutants), lead and mercury, among others.	
		Directive 2011/65/EU of the European Parliament and of the Council of 8 June 2011 on the restriction of the use of certain hazardous substances in electrical and electronic equipment, 2011.	Recast of Directive 2002/95/EC. Restriction of hazardous substances was expanded to other types of EEE. Defines 0.1% *w*/*w* (1000 ppm) as the admissible threshold values for the concentrations of PBDEs and PBBs that can be present in new products manufactured to be marketed in the EU.	
United Kingdom	1586	WEEE Directive (2013)	Implements Directive 2012/19/EU.	N/A

**Table A2:** Africa (Generalities).

Legislation is either Lacking or not Being Properly Enforced in These Countries. Regulation of WEEE Management Relies on International Agreements		
International Agreement		Reference
Basel Convention, 1992	International Environmental Agreement between 53 signatory countries aimed at controlling trans-boundary movements (import/export) of hazardous waste with specific restriction on the movement of toxic waste from developed to less developed or developing countries. Does not regulate WEEE directly but through the fact that WEEE contains hazardous substances and is therefore, hazardous waste. Allows hazardous waste movements if there is a bi/multi-lateral agreement for sound treatment of the waste in the destination country.	[57]
Bamako Convention, 1991 (enforced in 1998). African treaty.	Aimed at restricting hazardous waste movements into and between African countries. Does not mandate compliance but provides advice on management. Seeks to complement the requirements of the Basel Convention in order to prevent transboundary movement of hazardous waste into African countries. Creates a framework for adequate management of hazardous waste ensuring the protection of African communities from the negative health and environmental impacts of unregulated waste management.	

**Table A3:** Africa.

Generated Volume: 2.9 Mt Generated in 2019 [22]				
Country	WEEE Generation (kt/year)	Legislation/Regulation	Comments	Reference
South Africa	416	National Waste Management Strategy	Classifies WEEE as hazardous waste.	[58]
		EPR Regulations, 2020	The WEEE notice includes expected targets for collection and recycling over the following five years. Amendments to be proposed by May 2021.	
Nigeria	461	Harmful Waste (Special Criminal Provisions)	Prohibition of deposition and dumping of harmful waste on any land, territorial waters and related matters.	[59]
		National Environmental (Sanitation and Waste Control) Regulation 2009	No person is to engage in any activity likely to generate Hazardous waste without permission from the agency. That who generates hazardous waste must ensure such waste is treated using appropriate methods. Export and transit of hazardous waste is prohibited unless express permission from the agency. Hazardous waste destined to another country is not permitted to transit through Nigeria without consent of the agency.	
		Guide for Importers of Used EEE (UEEE)	Importers of UEEE to register with authority. Import of functional UEEE is allowed. Import of near-end-of-life WEEE is prohibited.	
		The National Environmental (Electrical Electronic Sector) Regulations SI No 23, 2011	Adopts polluter pays principle. Ensures environmentally sound waste management. Defines the roles and responsibilities of stakeholders.	

**Table A4:** Asia.

Generated Volume: 24.9 Mt Generated in 2019 [22]				
Country	WEEE Generation (kt/year)	Legislation/Regulation	Comments	Reference
China	10,129	Notification on seventh category waste importation, 2000	Import of the seventh category of waste is banned.	[60]
		Technical policy and pollution prevention and control of WEEE, 2006	Establishes the “Reduce, Reuse, Recycle” and the “polluter pays” principles. Designates resources for environmentally sound collection, reuse, recycling, and disposal of WEEE.	
		Prevention and Control of Pollution from IT Products, 2007	Restricts the use of hazardous substances. Sets eco-design requirements. Manufacturers/importers (producers) must provide information about their products.	
		Pollution Prevention of Waste Electrical and Electronic Equipment, 2008	Aimed at preventing pollution generated during disassembling, recycling and disposing of WEEE. Establishes a licensing scheme for WEEE recycling companies.	
		Regulation on recycling and disposal of WEEE, 2011	Defines an obligation to disassembly WEEE and centralize its recycling. Establishes a fund to support WEEE recycling.	
		Administrative measures for levy and use of treatment fund for waste electronic and electric products, 2012	Imported EEE must pay the fund The fund for WEEE treatment subsidizing is set by the state.	
		Restriction of Hazardous Substances in EEE, 2016	Introduces a compliance list for management. Establishes a system for conformity assessment.	[61]
		WEEE Treatment List, 2014 ed. Enforced in 2016	Extended the original 5 categories to 14.	
		WEEE fund standard update, 2016	Lowers the subsidy for management of waste TVs and PCs (personal computers), rises subsidy for waste air conditioners management.	
		Pilot project on EPR (extended producer responsibility) in electronics industry, 2015	Producers must lead EEE design and production, as well as WEEE collection and recycling.	
		Promotion Plan: EPR principle	Provides guidance for producers to implement eco-design, selection of secondary materials and involvement in WEEE collection and recycling.	
India	3230		Only authorized dismantlers and recyclers are allowed to collect WEEE.	[22]
		E-waste (management) Rules, 2016	Puts producer responsibility organization (PRO) in place.	
		National Resource Policy, 2019 (DRAFT)	Proposes a stronger role for producers regarding recovery of secondary resources from WEEE.	

**Table A5:** North America.

Generated Volume: 9.3 Mt Generated in 2019				
Country	WEEE Generation (kt/year)	Legislation/Regulation	Comments	Reference
United States of America	6918	No federal legislation regulating WEEE management in place.	Legislation varies between states: different scopes, impacts and bans on WEEE disposing in landfills. In general, all state which have implemented regulation take an EPR approach.	[22]
Mexico	1220	General Law for the Prevention and Integral Management of Waste (LGPEGIR—for its acronym in Spanish), 2004	Defines WEEE as technological waste and classifies it as special management waste, making states and municipalities responsible for its prevention, transport, storage, handling, treatment, and final disposal.	[62]
		NOM-161-SEMARNAT-2011	Sets the obligation to present plans for electrical and electronic waste was generated.	
		NADF-019-AMBT-2018 (Environmental Standard for the Federal District. Electrical and Electronic Waste, requirements and specifications for its management)	Seeks to establish the requirements and specifications for the correct separation, storage, collection, collection, transport, treatment, recycling and disposal of electrical and electronic waste within Mexico City.	
Canada	757	Ministry of Environment is responsible for WEEE management. No related federal legislation is in place. WEEE mainly managed under the Stewardship Programme: Electronic Product Stewardship Canada (EPSC) organized and controlled by the private sector.		[57]

**Table A6:** South America.

Generated Volume: 3.8 Mt Generated in 2019 [22]				
Country	WEEE Generation (kt/year)	Legislation/Regulation	Comments	Reference
Brazil	2143	National Solid Waste Policy	Establishes that every stakeholder within the lifecycle of EEE is responsible for its management at the end-of-life of the product. It promotes WEEE reverse logistics.	[22]
